# Prediction of Milk Coagulation Properties and Individual Cheese Yield in Sheep Using Partial Least Squares Regression

**DOI:** 10.3390/ani9090663

**Published:** 2019-09-07

**Authors:** Massimo Cellesi, Fabio Correddu, Maria Grazia Manca, Jessica Serdino, Giustino Gaspa, Corrado Dimauro, Nicolò Pietro Paolo Macciotta

**Affiliations:** 1 Dipartimento di Agraria, Sezione di Scienze Zootecniche, Università degli Studi di Sassari, Viale Italia 39, 07100 Sassari, Italy (M.C.) (M.G.M.) (J.S.) (C.D.) (N.P.P.M.); 2 Dipartimento di Scienze Agrarie Alimentari e Forestali, Università di Torino, 10095 Grugliasco, Italy

**Keywords:** clotting properties, individual cheese yield, mid-infrared spectroscopy, partial least square regression, sheep

## Abstract

**Simple Summary:**

Considered that all sheep milk in Italy is destined for cheese processing, traits describing rennet coagulation aptitude should be among the most important selection goals for dairy breeds. To reduce the costs and logistics related to the large-scale recording of these traits, mid-infrared (MIR) spectroscopy could be conveniently used to generate reliable predictions without any additional cost. The aims of this research were to predict the milk coagulation properties (MCP) and individual cheese yield (ILCY) in sheep by MIR spectrometry using partial least squares regression (PLS), and to compare different data pre-treatment procedures. The prediction results observed in the present study, although moderate, suggest the possibility of adding novel phenotypes (e.g., MCP and ILCY) in breeding schemes for dairy sheep breeds. Mid-infrared spectroscopy coupled with PLS regression could allow the prediction of phenotypes at the population level without additional costs.

**Abstract:**

The objectives of this study were (i) the prediction of sheep milk coagulation properties (MCP) and individual laboratory cheese yield (ILCY) from mid-infrared (MIR) spectra by using partial least squares (PLS) regression, and (ii) the comparison of different data pre-treatments on prediction accuracy. Individual milk samples of 970 Sarda breed ewes were analyzed for rennet coagulation time (RCT), curd-firming time (k20), and curd firmness (a30) using the Formagraph instrument; ILCY was measured by micro-manufacturing assays. An Furier-transform Infrared (FTIR) milk-analyzer was used for the estimation of the milk gross composition and the recording of MIR spectrum. The dataset (n = 859, after the exclusion of 111 noncoagulating samples) was divided into two sub-datasets: the data of 700 ewes were used to estimate prediction model parameters, and the data of 159 ewes were used to validate the model. Four prediction scenarios were compared in the validation, differing for the use of whole or reduced MIR spectrum and the use of raw or corrected data (locally weighted scatterplot smoothing). PLS prediction statistics were moderate. The use of the reduced MIR spectrum yielded the best results for the considered traits, whereas the data correction improved the prediction ability only when the whole MIR spectrum was used. In conclusion, PLS achieves good accuracy of prediction, in particular for ILCY and RCT, and it may enable increasing the number of traits to be included in breeding programs for dairy sheep without additional costs and logistics.

## 1. Introduction

Sheep cheese (680,302 tonnes) represents about 3% of the total world cheese production [1]. Mediterranean countries, where a large proportion of dairy sheep are farmed [2], produce about 45% of the world’s sheep cheese [3]. Italy is the fifth world producer in terms of quantity (7.5%), and it accounts for 36% of the world sheep cheese trade [4].

The Sarda breed, consisting of about 3.3 million heads, represents 60% of the Italian dairy sheep stock, and accounts for 80% of the total sheep milk produced in Italy [4]. All the milk is destined to cheese production with the manufacturing of three Protected Designation of Origin cheeses: Pecorino Romano, largely exported to North America (more than 6000 t/yr) [5], Pecorino Sardo, and Fiore Sardo. The current breeding goals for the Sarda breed (220,268 ewes recorded in 2014) [6] are total lactation milk yield and scrapie resistance. Milk fat and protein contents are measured routinely only on first and second lactation ewes on a limited number of official tests. 

As all sheep milk is destined for cheese processing, technological properties should be among the most important selection goals for dairy breeds. Actually, most sheep programs select only for total milk yield per lactation, and major milk components are recorded in only a few cases, i.e., fat and protein contents [7]. The main reason is the cost of milk performance recording, which is usually higher in sheep than in cattle. On the other hand, consumer concerns about food quality, the specific requirements of the cheese industry, and the large number of variables that could be currently measured by high throughput phenotyping systems represent strong elements in favor of the introduction of these traits in the selection plans of dairy breeds.

The cheese-making properties of milk are defined by several variables that are connected by a complex net of correlations [8,9]. Apart from fat and protein, which are the most important milk constituents affecting cheese yield, milk coagulation properties (MCP) are popular phenotypes for describing individual milk technological quality [10]. In particular, rennet coagulation time (RCT, min), curd-firming time (k20, min), and curd firmness (a30, mm) are attracting attention as indicators of milk cheese-making aptitude in dairy cows [11,12]. Another phenotype that has been proposed for describing milk technological properties is individual laboratory cheese yield (ILCY), which is obtained by micro-manufacturing assays [9,13].

In ewes, MCP are affected by the chemical and physical characteristics of milk, such as fat and protein contents, somatic cell count, pH, and temperature, as well as by some environmental factors, such as lactation stage, lambing season, and flock test date [14,15]. Furthermore, a number of works on dairy cattle [16,17] and, recently, on dairy sheep [18] have evidenced genetic variability for MCP, thus suggesting the possibility to select for milk cheese-making ability.

The inclusion of both MCP and ILCY in milk-recording performance programs appears to be rather problematic because of the increase of costs and logistics. However, mid-infrared (MIR) spectroscopy, which is widely used in milk routine analysis (mainly for fat, protein, and lactose measurements), could be used to generate proxies of milk quality traits without any additional cost [19].

The main issue in using MIR spectra for prediction purposes is the large number of predictors—more than 1000—and the large degree of correlation between them. The partial least squares regression (PLS) is a multivariate regression technique commonly used for relating MIR spectra to milk phenotypes (e.g., MCP), and to build prediction models for the investigated traits. This technique is able to reduce the predictor space by extracting a lower number of predictors that are uncorrelated. In particular, PLS has been used to predict coagulation properties in dairy cattle [20,21]. In order to achieve good accuracies of prediction, spectra variable selection and mathematical pretreatments have been used, giving different results, depending on the method used to build the equations [22], but also on the considered traits [23]. The LOWESS (locally weighted scatterplot smoothing) regression is a procedure that operates separate regressions in different intervals, into which the dependent variable is fragmented, with the aim to remove the noise from data patterns. This technique is not of frequent use in animal science, in general, but it is effective in smoothing irregular patterns (such as, for example, the signal of the Fisher fixation index along the genome [24]).

MCP prediction have been extensively investigated in dairy cattle, whereas few works have been conducted on the same issue in dairy ewes. Although cheese yield generally represents one of the most important economic traits for the dairy industry, there is a lack of information on the MIR prediction of this trait for ovine milk. The prediction of cheese yield, with reasonable accuracy, would be of crucial importance for the ovine sector, particularly in Italy, where almost all sheep milk is destined for cheese production. This lack of information could arise from the difficulty of measuring cheese yield in a high number of samples. Laboratory cheese yield needs a small volume of milk (10 mL), and could provide an indicator of the efficiency of the cheese-making process.

The aims of this research were to predict milk coagulation properties and individual cheese yield in sheep by mid-infrared (MIR) spectrometry using partial least squares regression and to compare different data pre-treatment procedures (i.e., the use of raw or reduced MIR spectra and the use of the LOWESS procedure).

## 2. Materials and Methods

### 2.1. Animals and Sampling

A total of 970 individual sheep milk samples were collected by the Provincial Association of Breeders (APA) in the period from April to July 2014 from 47 farms distributed in the four historical provinces of Sardinia (Cagliari, Nuoro, Sassari and Oristano, Italy). Milk samples, after preservative addition (bronopol, 62.5 μL/100 mL), were divided into two aliquots. One aliquot was used to generate the MIR spectrum; the other was destined to the measurement of milk technological properties. 

Milk samples were collected as part of routine data and sample collection, during the breeding program operated by the Provincial Breeders Farmers Associations (APA) of Cagliari, Nuoro, Sassari and Oristano (Sardinia, Italy). For this reason, it was not necessary to obtain permission from the ethics committee.

### 2.2. MIR Spectra

The MIR spectrum of each milk sample was generated by a MilkoScanFT6000 equipment (Foss Electric, Hillerød, Denmark) during routine milk composition analysis performed in the milk laboratory of the Regional Association of Animal Breeders of Sardinia (ARAS, Oristano, Italy). MIR spectra were recorded in the region between 5011.54–925.92 cm^−1^. Since instrumental resolution is 3.858 cm^−1^, each spectrum consisted of 1060 data points.

### 2.3. Analysis of Milk Technological Properties

The analysis of MCP was performed by using the Formagraph instrument (Foss Electric A/S, Hillerød, Denmark) according to Zannoni and Annibaldi [25]. The following three parameters were recorded: rennet coagulation time (RCT), curd-firming time (k20) and the curd firmness at 30 min (a30). Samples that did not coagulate within 30 min were excluded (n = 111).

The individual laboratory cheese yield was measured according to the method of Othmane et al. [13] with some modifications as previously described by Manca et al. [9]. Briefly, 10 g of raw milk samples, heated at 40 °C, were exactly weighed into test tubes (15-mm internal diameter) and then equilibrated at 36 °C for 10 min in a water bath. A volume of 40 μL of the rennet solution was added to the milk samples in the tubes, reaching a final dose of 0.060 IMCU/g. The rennet work solution (15 IMCU/mL) was prepared, freshly, by diluting 1000 IMCU/mL 100% chymosin solution (CHY-MAX ^®^ M 1000 Hansen A/S, Denmark) in ultra-pure water. The tubes were closed and quickly inverted, to ensure uniform distribution of the rennet, and kept at 36 °C for 1 h in a water bath. Then, the formed coagulum was cut (into the tube) with a cutter in the form of a cross and centrifuged at 4000 rpm for 15 min at 36 °C, in order to separate curd from whey. The whey was removed by draining for 45 min with the test tube facing downwards. ILCY was expressed in % (w/w) of the relative weight of the centrifuge residue on the initial weighed milk.

The method used was chosen as it was relatively easy to perform on a large number of samples with a small volume of milk. The method was also used by other works and gave reasonable correlation [18] with the cheese yield obtained with the equation of Pirisi et al. [26], which was used to predict the Pecorino Romano cheese yield from bulk milk composition. 

### 2.4. Statistical Analysis

The dataset (n = 859) was divided into two sub-datasets: the first consisted of 700 ewes, and it was used for estimating the parameters of the prediction model (EST); the remaining 159 animals were used to validate the model (VAL). In order to account for sampling effect, 100 replicates were performed, assigning animals randomly to the EST and VAL datasets, respectively.

PLS was used to predict the MCP and ILCY from MIR spectra using the PLS procedure of SAS/STAT software version 9.4 [27]. Expressed in matrix notation, the PLS model is:

Y = XB + E,(1)

where Y= (y_1_, y_2_, …, y_m_) is the matrix of m response variables (in the case of the present work, m = 4: RCT, k20, a30, ILCY) measured on *n* subjects (700 ewes of the EST dataset); X = (x_1_, x_2_, …, x_p_) is the matrix of *p* predictors (the MIR spectra according to the four scenarios described above) measured on the same *n* subjects; B = is the matrix of regression coefficients to be estimated; and E = is the matrix of residuals.

The B matrix estimated in the EST dataset was used to make predictions for the VAL dataset. Based on preliminary runs of PLS and to the PRESS (predicted residual error sum of squares) statistics, the number of latent variables to extract from the predictors was set to 34.

Prediction was carried out by using the whole MIR spectra (all_MIR), or the reduced spectra (red_MIR) from which regions corresponding to wave number of water absorption (from 3105 to 3444 cm^−1^ and from 1628 to 1658 cm^−1^) were removed [28]. Moreover, spectral data were analyzed directly or after a correction carried out using a locally weighted scatterplot smoothing (LOWESS). The LOWESS is a smoothing procedure that performs separate regressions in different intervals into which the dependent variable is fragmented. Aims of the LOWESS are the removal of noise from data patterns. In the present study, this analysis was performed using the PROC LOWESS of SAS/STAT software version 9.4 [27]. The smoothing parameter defines the width of each interval of the dependent variables (in this case, the wavelength) into which PLS fits separate regressions, which we set to 0.03. In a preliminary test, we carried out the analysis using the common first derivative treatments in our data, but smoothing performed better (only on the whole spectrum).

Four scenarios of prediction were compared in the validation step: (1) whole_MIR spectrum and raw data (all_MIR_raw); (2) whole_MIR spectrum and LOWESS corrected data (all_MIR_LOWESS); (3) reduced_MIR spectrum and raw data (red_MIR_raw); (4) reduced_MIR spectrum and LOWESS corrected data (red_MIR_LOWESS).

Prediction accuracy was assessed by calculating the coefficient of determination R^2^ between observed and model predicted values, the residual mean squared error of prediction (RMSEP), and the slope between observed and predicted values in VAL animals.

## 3. Results and Discussion

The mean values of milk composition, MCP, and ILCY are reported in Table 1.

Values are in agreement with previous reports on Sarda dairy ewes [15,29]. As far as MCP are concerned, the average RCT is similar to the values observed in the Sarda [30,31] and Spanish [32,33] breeds, but it is larger than a recent report on Sarda ewes [15]. Curd-firming time and curd firmness show values similar to previous reports for dairy sheep [15,34]. The average ILCY is quite large: higher than values observed in Churra ewes [13], but quite similar to those reported in Merino ewes [35]. Of course, these laboratory values are quite far from the cheese yield observed in industrial plants; i.e., the value of ILCY observed in the present study doubles reported values for Pecorino Romano cheese [36]. As pointed out by Manca et al. [9], such differences could arise from the peculiarity of micro-manufacturing experiments, which need a smaller amount of milk to be processed and a different method of curd drying, compared with an industrial cheese-making process.

Prediction statistics (Table 2) were generally moderate, and in agreement with previous reports for dairy cattle [20,37]. The largest coefficient of determination values were observed for ILCY (0.64 averaged across all scenarios) and RCT (average 0.55), whereas the smallest were obtained for k20 (average 0.42) and a30 (average 0.36), respectively. 

Cheese yield represents generally the most important economic trait for the dairy industry and, indirectly, for the definition of price of milk [21]; this is of crucial importance for the ovine sector, particularly in Italy, where almost all sheep milk is destined for cheese production. Despite this, the MIR prediction of this trait has been scarcely investigated in ovine milk, making comparison with literature difficult. Similar results to those found in the present work, in terms of the calibration R^2^ value and slope of regression between observed and predicted values, were reported in a previous study on dairy cattle [38], using a reduced MIR spectrum and the PLS method. The higher prediction accuracy that we found for ILCY compared to RCT was in agreement with that work.

Considering the importance of cheese yield for the economy of dairy industries, the inclusion of the only MCP in the breeding program should be considered carefully. In fact, MCP exhibited moderate correlation with ILCY in the study of Manca et al. [9], and similarly, Bonfatti et al. [11] found a very weak or trivial association of MCP with cheese yield. Thus, the importance of predicting cheese yield becomes important in a milk payment system that aims to improve the cheese yield.

The average R^2^ values for MCP were partially in agreement with a previous work carried out on Sarda dairy ewes using a Bayesian model [39] and, for RCT, with reports on grazing dairy cows [40]. 

The prediction ability obtained in the present work was also very similar to that observed in a recent investigation on cattle [37]. The lowest value of prediction for a30 observed in the present study was consistent with a previous investigation on cattle, where this trait exhibited a lower prediction accuracy in comparison with the other MCPs [20]. In general, these results confirm the feasibility, for ovine milk as well, of MIR spectroscopy to carry out analysis, rapidly and without additional costs, considering that it can be performed directly to the samples collected during the routinely milk recording. This is important when the phenotypes are thought to be included in a breeding program, as a large individual dataset is needed.

The ILCY showed the best values (i.e., close to one) of the slope between predicted and observed values (Table 2). Also, the intercept between observed and predicted was in most cases not significantly different from zero, indicating an absence of systematic bias. The worst values were observed for a30. 

**Figure 1:**
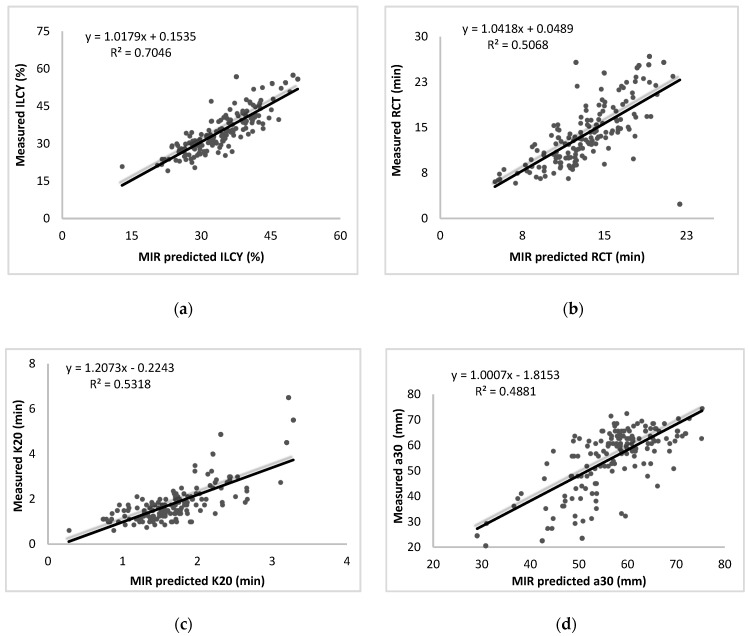
Partial least squares (PLS)-predicted individual laboratory cheese yield (ILCY) (**a**), rennet coagulation time (RCT) (**b**), curd-firming time (k20) (**c**), and curd firmness at 30 min (a30) (**d**), using the reduced MIR spectrum without locally weighted scatterplot smoothing (LOWESS) correction plotted against observed values.

The plot of observed versus MIR-predicted traits in the red_MIR_raw scenario for one of the 100 replicates is reported in Figure 1. In the case of ILCY, the model tends to underestimate this trait for large values (Figure 1a). This tendency can be observed also for RCT (Figure 1b) and, markedly, for curd-firming time (Figure 1c). The predictions for a30 are characterized by a larger dispersion of data (Figure 1d).

The use of the reduced MIR spectrum yielded better results for all the four considered traits (Table 2). On average, predictions based on the reduced spectrum exhibited an R^2^ of 6% higher in comparison with the use of the entire spectrum. A similar pattern could be observed for RMSEP: in general, lower values (desirable) were observed when the reduced MIR spectrum was used. These results are consistent with previous investigations where the discharge of a portion of MIR spectrum before PLS analysis is recommended to obtain better prediction models [41].

**Figure 2:**
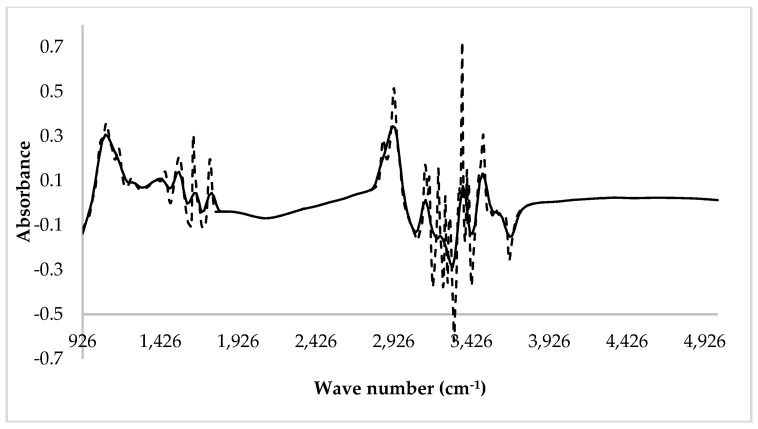
Raw (dotted line) and LOWESS corrected (solid line) MIR milk spectrum of an ewe.

The LOWESS correction improved prediction ability only when the complete MIR spectrum was considered. No effects of data smoothing were detected when the reduced spectrum was used. The reason can be inferred by looking at Figure 2, which reports both the raw and the LOWESS corrected spectrum of one ewe.

The use of other data correction techniques has been previously reported, evidencing contrasting results [20,42]. In fact, it has been demonstrated that the accuracy of prediction can depend on several factors, such as the investigated variable, the population studied, the spectra variable selection, and mathematical pre-treatments [22,38]. Recently, Bayesian models have been also evaluated with the aim to improve the accuracy of infrared prediction equations of different traits, including technological properties of bovine and ovine milk [38,39]. The improved prediction accuracies observed for MCP were not confirmed in other studies that compare PLS and Bayesian methods of regression, which observed null or negligible differences in prediction accuracy between the two approaches—PLS and Bayesian—after reducing noise by spectral mathematical treatments [22,43]. It was stressed that, depending on the method used to build the equations, spectra variable selection and mathematical pre-treatments can influence the accuracy of prediction models [22].

The PLS analysis gave better prediction for RCT using untreated data compared to pre-treatments as normalization, multiplicative scatter corrections, or the use of first and second derivatives, whereas, pre-treatment yielded better predictions for other traits (i.e., titratable acidity and pH) [20].

The results obtained in the present work, in terms of accuracy of prediction, in particular for ILCY, deserve to be further investigated. For example, an evaluation using an external dataset for the validation procedure should be carried out. As far as the choice of the validation procedure is concerned, different approaches have been used by authors of previous works: full cross-validation [20,44], validation using an external dataset, or both [23,37,40]. Some authors stated that the use of an external dataset should be more appropriate for the validation of the calibration procedure based on FTIR spectra [39]. On the other hand, the use of the entire dataset increases the size and the variability of the sample, giving better accuracies of prediction. In the present work, we chose to use all data available and assign animals to EST and VAL data randomly. The rationale of this choice was to mimic possible large-scale applications where large database made by animals of different flocks are likely to be used; phenotypes of animals from different flocks are predicted as well.

## 4. Conclusions

Mid-infrared spectroscopy could allow the prediction of phenotypes at the population level without additional costs. Although moderate, the prediction results obtained in the present work suggest the possibility of adding novel phenotypes in breeding schemes for dairy sheep breeds. In particular, the PLS regression was able to yield predictions for individual cheese yield and rennet coagulation time with reasonable accuracy. Considering the importance of cheese yield in the definition of profitability of dairy industries, and the unfavorable economic condition of the dairy sheep sector in Italy [45], this phenotype (which can be predicted, inexpensively, at the population level) should be taken into account in the selection strategies for an improved cheese-making aptitude of Sarda sheep milk.

## Figures and Tables

**Table 1:** Average values for milk composition traits, milk coagulation properties, and individual cheese yield of the considered sample of ewes.

Trait ^1^	Mean	Sd
Milk yield (kg/d)	1.72	0.43
Fat content (%)	6.01	1.33
Protein content (%)	5.43	0.58
Lactose content (%)	4.86	0.28
SCC (×1000 cells/mL)	883,000	2389
pH	6.55	0.11
RCT (min)	13.45	4.43
k20 (min)	1.73	0.07
a30 (mm)	55.67	11.59
ILCY (%)	35.20	8.20

^1^ SCC: somatic cell count; RCT: rennet coagulation time; k20: curd firming time; a30, curd firmness; ILCY: individual cheese yield.

**Table 2:** Statistics of partial least square predictions for milk coagulation properties and individual laboratory cheese yield in the validation dataset averaged for 100 replicates.

Trait ^1^	Scenario ^2^	R ^2,3^	RMSEP ^4^	b_obs,pred_^5^	a_obs,pred_^6^
ILCY (%)	All_MIR_Raw	0.60 ± 0.05	5.19 ± 0.40	0.92 ± 0.07	2.67 ± 2.26
	All_MIR_LOWESS	0.65 ± 0.05	4.95 ± 0.38	0.95 ± 0.07	1.65 ± 2.39
	Red_MIR_Raw	0.66 ± 0.05	4.78 ± 0.38	0.96 ± 0.07	1.36 ± 2.38
	Red_MIR_LOWESS	0.66 ± 0.05	4.81 ± 0.38	0.95 ± 0.07	1.77 ± 2.44
RCT (min)	All_MIR_Raw	0.49 ± 0.07	3.15 ± 0.25	0.87 ± 0.09	1.71 ± 1.17
	All_MIR_LOWESS	0.53 ± 0.08	3.02 ± 0.26	0.90 ± 0.13	1.30 ± 1.68
	Red_MIR_Raw	0.59 ± 0.10	2.81 ± 0.35	0.91 ± 0.16	1.23 ± 2.10
	Red_MIR_LOWESS	0.59 ± 0.09	2.83 ± 0.33	0.91 ± 0.13	1.18 ± 1.18
k_20_ (min)	All_MIR_Raw	0.37 ± 0.07	0.54 ± 0.06	0.79 ± 0.12	0.36 ± 0.20
	All_MIR_LOWESS	0.41 ± 0.06	0.52 ± 0.06	0.86 ± 0.13	0.24 ± 0.21
	Red_MIR_Raw	0.47 ± 0.07	0.49 ± 0.05	0.88 ± 0.12	0.20 ± 0.20
	Red_MIR_LOWESS	0.43 ± 0.07	0.51 ± 0.05	0.86 ± 0.14	0.24 ± 0.22
a_30_ (mm)	All_MIR_Raw	0.31 ± 0.05	9.60 ± 0.56	0.77 ± 0.09	12.5 ± 5.64
	All_MIR_LOWESS	0.32 ± 0.06	9.51 ± 0.57	0.83 ± 0.11	9.33 ± 6.56
	Red_MIR_Raw	0.42 ± 0.06	8.78 ± 0.52	0.87 ± 0.10	7.35 ± 5.81
	Red_MIR_LOWESS	0.38 ± 0.06	9.18 ± 0.55	0.84 ± 0.10	8.54 ± 6.00

^1^ ILCY: individual cheese yield; RCT: rennet coagulation time; k20: curd-firming time; a30, curd firmness. ^2^ All_MIR_Raw: entire MIR spectra without correction; All_MIR_LOWESS_MIR: entire mid-infrared (MIR) spectra smoothed by local regression technique; Red_MIR_Raw: MIR spectra without water regions absorptions and without correction; Red_MIR_LOWESS: MIR spectra without water region absorptions and smoothed by local regression technique. ^3^ R^2^: coefficient of determination. ^4^ RMSEP: root mean squared error of prediction. ^5^ b: obs, pred: regression coefficient between predicted and observed. ^6^ a: obs, pred: intercept between predicted and observed.
